# Surgical management of giant secondary malignant fibrous histiocytoma following radiotherapy for nasopharyngeal carcinoma: A case report and literature review

**DOI:** 10.3892/ol.2014.2069

**Published:** 2014-04-15

**Authors:** LIPING TONG, YONG WANG, YONGAN ZHOU, XIAOQING ZHENG, HONGGANG LIU, JIANYONG SUN, XIAOFEI LI, XIAOLONG YAN

**Affiliations:** 1 Department of Thoracic Surgery, Tangdu Hospital of the Fourth Military Medical University, Xi’an, Shaanxi 710038, P.R. China; 2 Department of General Surgery, Weinan Center Hospital, Weinan, Shaanxi 714000, P.R. China

**Keywords:** malignant fibrous histiocytoma, surgical treatment, radiotherapy

## Abstract

Malignant fibrous histiocytoma (MFH) is rare in the chest wall, particularly in patients who have undergone radiotherapy for primary nasopharyngeal cancer. In the present study, a case of MFH of the upper chest wall that appeared four years after initial radiotherapy for squamous cell carcinoma of the nasopharynx is reported. Furthermore, two-step surgical management was successfully performed consisting of i) tumor-reductive excision and ii) limb salvage surgery, including wide resection of the tumor mass, defect reconstruction of the chest wall using left latissimus dorsi myocutaneous flap and dermatoplasty of the flap-supplied region. The progress of the clinical characteristics, the reasons for radiation-induced carcinogenesis, the treatment options and the prognostic factors of MFH are also reviewed. Finally, the importance of prevention and follow-up of this malignancy are highlighted and specific advice is offered.

## Introduction

Malignant fibrous histiocytoma (MFH) is one of the most common soft-tissue sarcomas in adults (1) and has the aggressive clinical feature of a high rate of local recurrence, and a short survival time. MFH accounts for ~10% of all head and neck sarcomas (2), and the tumor size and depth, and the presence of regional lymph node metastases are significant risk factors affecting the survival rate (3).

Radiotherapy is an integral part of cancer management, with >50% of all patients undergoing radiation treatment (4), particularly in the head and neck region. However, it is well known that radiation can induce major side-effects, including radionecrosis and oncogenesis. A recent multicenter study showed that radiation-induced soft-tissue sarcomas accounted for 0.9% of 5,046 patients with soft-tissue sarcomas, and that the most common diagnosis is MFH (36.4%) (5).

To date, there have been numerous reported cases of secondary sarcoma subsequent to radiation therapy for nasopharyngeal carcinoma, but only one case of MFH of the sphenoid bone due to radiotherapy for nasopharyngeal carcinoma has been reported (6). In the present study, a case of MFH of the upper chest wall that appeared four years after initial radiotherapy for squamous cell carcinoma of the nasopharynx is described. In addition, the successful two-step surgical procedure is highlighted and the literature concerning MFH is reviewed. The patient provided written informed consent.

## Case report

A 28-year-old female was admitted to the Department of Thoracic Surgery of Tangdu Hospital (Xi’an, China) complaining of a fast-growing mass in the upper chest wall, which had been diagnosed as MFH by biopsy in another hospital. Subsequent to obtaining the medical history of the patient, the following information was established. In August 2007, the patient was diagnosed with poorly-differentiated squamous cell carcinoma of the nasopharynx due to durative nasal hemorrhage and diminishing hearing in the left ear. In view of the metastases of the cervical and supraclavicular lymph nodes, the patient did not undergo radical surgery. Therefore, wide irradiation (from the nasal tip to subclavicular region) was delivered in a total dose of 70 Gy in 35 fractions, followed by three courses of chemotherapy based on platinum (20 mg/m^2^ cisplatin combined with 500 mg/m^2^ 5-fluorouracil on days one to five). The patient then received regular medical examinations and was confirmed to be disease-free. In December 2011, a small mass with a diameter of 1 cm was detected in the subclavicular region and a tumor biopsy was carried out. According to the histological characteristics and immunohistochemisty results the patient was diagnosed with MFH. Staining for AE1/AE3, S-100, smooth muscle actin (SMA), desmin, cluster of differentiation 31 (CD31), CD34 and anaplastic lymphoma receptor tyrosine kinase (ALK) was negative, while the staining for vimentin was strongly positive (+++) with >50% positive tumor cells. A Ki-67 of >30% was detected. In fear of possible amputation of the left upper extremity, the patient refused surgical treatment and underwent targeted cryoablation therapy using argon and helium. The tumor grew more slowly until January 2013. Within the next month, the tumor mass was enlarged to 15×15×5 cm, and the tumor surface began to ulcerate and bleed and became infected. In less than a three-month period prior to being admitted to the Department of Thoracic Surgery of Tangdu Hospital (Xi’an, China), the patient experienced two emergency treatments due to hemorrhagic shock and an unmanageable infection.

**Figure 1:**
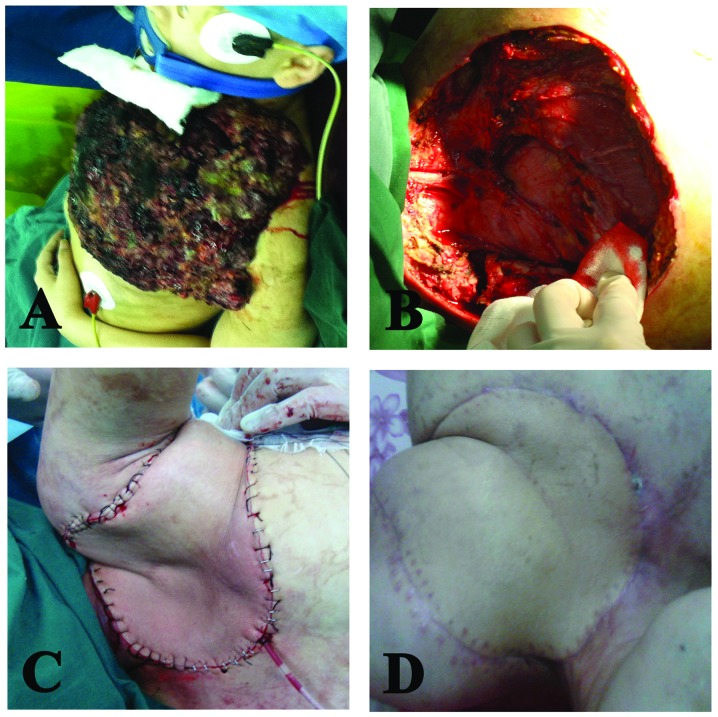
(A) Mushroom-shaped mass (20×20×6 cm) in the left anterior-superior chest wall, with the odor of necrotic tissue. (B) Sugical defect measuring 22×22×8 cm following excision of the whole tumor mass, the left clavicle, part of the first rib, the affected sternum and the sternoclavicular joint. (C) Defect reconstruction using left latissimus dorsi myocutaneous flap. (D) Two weeks after the surgery.

**Figure 2:**
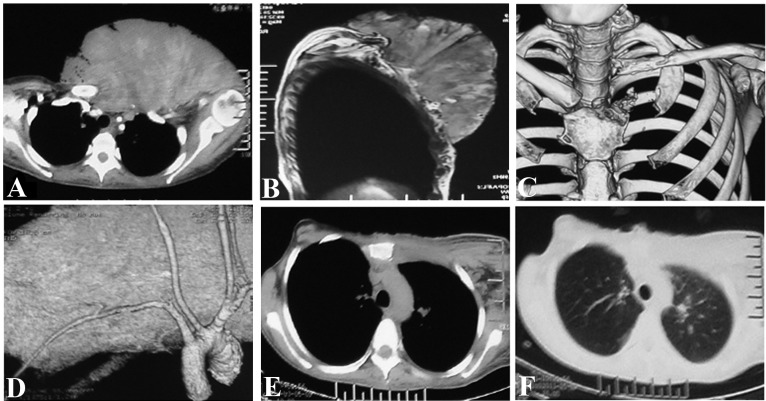
(A) Computed tomography (CT) and (B) magnetic resonance imaging showing an expansive mass in the left anterior-superior chest wall with an irregular margin, but with no evidence of invasion of the pleural cavity. (C) Three-dimensional imaging of the thoracic cage showing tumor invasion of the left clavicle, the first rib, superior segment of the sternum and the sternoclavicular joint. (D) The CT angiography indicated that the main blood supply of the tumor came from the proximal end of the left subclavian artery, particularly the internal thoracic artery. (E and F) The thoracic CT was rechecked and no signs of a remaining tumor were detected.

Following hospitalization, an integral clinical examination was performed within our institution. Upon physical examination, a mushroom-shaped mass (20×20×6 cm) that had the odor of necrotic tissue was found in the left anterior-superior chest wall (Fig. 1A). The laboratory examination indicated an exceptional white blood cell count (65.13×10^9^/l) and a slightly decreased hemoglobin concentration (106 g/l, post-transfusion with 10.0 units red blood cells one week earlier). The magnetic resonance imaging (MRI) and computed tomography (CT) plus three-dimensional imaging of the thoracic cage showed an expansive mass in the left anterior-superior chest wall, with an irregular margin invading the left clavicle, the first rib, the superior segment of the sternum and the sternoclavicular joint. However, there was no evidence of invasion of the pleural cavity (Fig. 2A–C) and no signs of systematic metastasis. The CT angiography and interventional angiography indicated that the main blood supply of the tumor came from the proximal end of the left subclavian artery, particularly the internal thoracic artery (Fig. 2D).

To improve the systematic condition for improved tolerance of the following surgery, the patient was administered specific necessary supportive care, including albumin prepared from human plasma, antibiotics, hemostatics, a blood transfusion and enteral nutrition. Nonetheless, the patient remained so weak that the development of a two-step surgical strategy was required. At first, tumor-reductive surgery, in which a tumor mass weighing >4 kg was excised, was performed. Following this, the systematic condition was further favored through the aforementioned methods. Nine days later, the secondary limb-sparing surgery was performed that included resection of the whole tumor mass, the left clavicle, part of the first rib, the affected sternum and the sternoclavicular joint, with a wide margin beyond 2 cm from the tumor (Fig. 1B). The surgical defect in the chest wall was then reconstructed using a left latissimus dorsi myocutaneous flap (Fig. 1C and D), and dermatoplasty in the left back was conducted using autologous skin at a thickness of 0.2 mm, obtained from the homolateral thigh of the patient.

**Figure 3:**
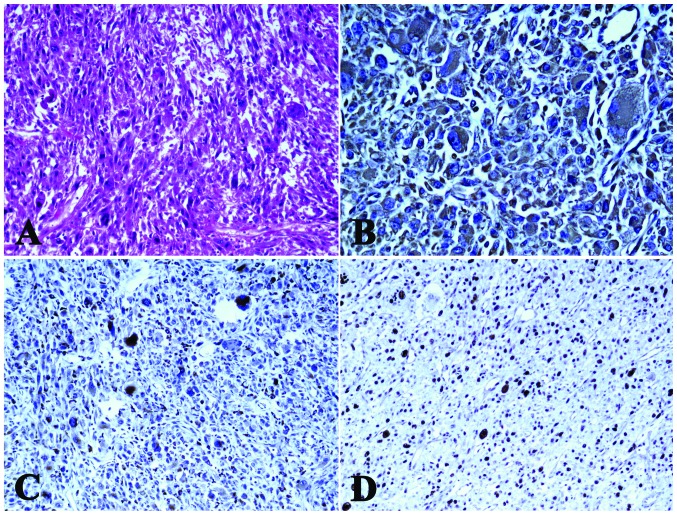
(A) High-power photomicrograph showing proliferation of pleomorphic spindle-shaped cells woith large irregular nuclei with hyperchromasia. A giant cell is shown in the center of the field, with sparse lymphocytes. Photomicrographs of the tumor with immunohistochemical staining: (B) Vimentin demonstrating a strongly positive reaction in the tumor cells. (C) CD68 demonstrating a weakly positive reaction in the tumor cells. (D) Ki-67 (MIB-1) demonstrating a positive reaction in the tumor cells. The MIB-1 labeling index was 30%. CD68, cluster of differentiation 68.

The mass was fixed in formalin, embedded in paraffin and underwent histological and immunohistochemical staining according to the standard methods. Hematoxylin and eosin-stained sections showed the presence of a malignant neoplasm composed of spindle-shaped cells, with an eosinophilic cytoplasm and scattered, abnormal mitotic figures. The tumor cells showed marked cellular pleomorphism, with unusually large and irregularly-shaped nuclei (Fig. 3A). The tumor cells were strongly and diffusely positive for vimentin (Fig. 3B) and CD68 (Fig. 3C). These spindle cells were weakly and focally positive for Ki-67 (MIB-1). Additionally, the MIB-1 labeling index was 30% (Fig. 3D). However, the tumor cells were negatively stained for myoglobin, SMA, desmin and S-100. The final pathological diagnosis was of MFH.

The post-operative course was uneventful. The thoracic CT was rechecked and no signs of a remaining tumor were detected. The patient was discharged from the hospital following a 49-day stay.

## Discussion

MFH is the most common type of mesenchymal tissue sarcoma in middle-aged or elderly adults, frequently arising from the proximal end of the extremities and in the retroperitoneum (1). MFH originating from the thoracic wall is the fifth most uncommon sarcoma (7), and post-irradiation MFH, which is mainly followed by radiotherapy for breast cancer, is even more infrequent than primary MFH. To the best of our knowledge, there has only been one reported case of MFH of the sphenoid bone due to radiotherapy for nasopharyngeal carcinoma (6). In the present study, a case of MFH of the upper chest wall that appeared more than four years after initial radiotherapy for squamous cell carcinoma of the nasopharynx is described. In addition, subsequent to reviewing the medical history of the patient, a relatively steady period was found following targeted cryoablation therapy and rapid progression without distant metastasis, which improved our knowledge with regard to the clinical features of the irradiation-induced MFH.

Historically, MFH tumors consist of spindle-shaped fibroblastic cells and bizarre mononuclear histiocytic cells arranged in a storiform pattern (8). In other words, MFH has a potential for bilateral differentiation. MFH has been classified into five subtypes: Inflammatory, myxoid, giant cell, storiform-pleomorphic and angiomatoid MFH (8). These categories are rarely used nowadays, which is largely due to their proper classification as entirely different types of sarcoma, which was generated by utilizing advances in IHC. Furthermore, all the aforementioned categories are positive for vimentin (9). Immunopositivity for vimentin, α1-antichymotrypsin and Ki-67 has been demonstrated in MFH and is helpful for diagnosis (6). By contrast, the tumor tissue should be immunonegative for S-100 protein and cytokeratins. In addition, histiocytic markers (CD68, α1-antichymotrypsin and factor XIII) no longer play a definitive role in the diagnosis of MFH, as immunoreactivity to these markers has been found to be non-specific (10). In the present case, the tumor cells were positive for vimentin, CD68 and Ki-67, and negative for S-100, SMA, desmin, CD31, CD34, ALK and AE1/AE3. Therefore, the patient was unconditionally diagnosed with MFH.

Although the combination of histological characteristics and immunohistochemical results was useful in the diagnosis of MFH in the present case, a suspected diagnosis of secondary sarcoma remains. The criteria published by Cahan *et al* (11) formed the basis of the diagnosis of radiation-induced soft-tissue sarcoma. Criteria found in the present patient included previous radiation treatment for various types of tumors (benign or malignant), development of a sarcoma in the area that was irradiated, the presence of a sarcoma that was histologically different from the primary cancer and a minimum dormant period of five years between the initial radiation and the development of the sarcoma. A recent modification of this definition indicated that a dormant period of three years was adequate for the diagnosis of post-irradiation sarcoma (12). Accordingly, we believe that the MFH tumor was secondary to radiotherapy for primary nasopharyngeal squamous cell carcinoma in the present case.

Oncogenesis is a well-known multi-step process that involves multiple genetic changes and results in the transformation of normal cells into malignant cells. Ionizing radiation has long been recognized to have carcinogenic potential. The long-term follow-up of the A-bomb survivors in Hiroshima and Nagasaki provided certain conclusive evidence for radiation-induced secondary malignant neoplasm due to the increase in the incidence of leukemia and solid tumors (13). Several mechanisms have been proposed for the pathogenesis of radiation-induced secondary malignancies. First, gene mutations can occur following exposure to radiation. These include base damage and single- and double-stranded DNA breaks, which can then cause a malignant transformation of the irradiated cell (14). Secondly, impairment in the DNA repair proteins could lead to an increased susceptibility to radiation-induced carcinogenesis. For example, it has been observed that radiation doses of >0.2 Gy fail to activate the G_2_/M cell cycle check-point, which could result in failure to repair DNA damage and eventually carcinogenesis (15). Finally, another mechanism (16) is the radiation-induced tissue inflammation and bystander effect that involves intercellular communication through gap junctions and systemic cytokine signaling, which have significant roles in the process of carcinogenesis.

Following the diagnosis of MFH in the present study, further medical imaging investigations and physical examinations were performed to delineate the extent of the local invasion and to assess for distant metastasis. Due to the poor general condition of the patient, a two-step surgical proposal was offered. As expected, the patient successfully survived surgery and was rehabilitated well.

Surgery remains the preferential choice for the treatment of primary or secondary MFH, although it has a high rate of local recurrence (17). A recent study (5) documented that following surgical resection for radiation-induced soft-tissue sarcoma, the occurence rate of positive margins ranged from 17–46%, and the rate of local recurrence ranged from 26–65% among 42 patients. Wide local excision with a 2–3 cm margin has been the most commonly employed approach. However, obtaining negative margins always ensures that surgeons face extensive reconstructive challenges. Those patients with post-irradiation sarcomas often have soft-tissue fibrosis, which not only make it difficult to identify normal tissue planes and to determine the true extent of tumor intraoperatively, but also can affect wound healing following the surgery (18).

For those tumors in which clear margins cannot be obtained, radiotherapy is used as an adjuvant treatment in certain settings. In a large study (19), patients with MFH of the extremities who underwent limb sparing surgery followed by radiation experienced a 10-year relapse-free survival rate of 62% and an overall survival rate of 80%, which was higher than that in earlier studies. Traditionally, chemotherapy is employed only for widespread disease, but large trials have not shown a significant benefit (20). The potential use of the multiple tyrosine kinase inhibitor, sunitinib, for MFH is currently undergoing a phase II trial (21). Todoroki *et al* (22) reported the case of a long-term survivor of relapsed MFH of the thigh treated with an autologous tumor vaccine combined with limb-sparing surgery and radiotherapy. Although promising, the widespread use of molecular-targeted therapy for MFH appears to remain distant. Recently, the National Comprehensive Cancer Network guidelines stated that adjuvant therapy should be considered on an individual case basis (23).

Peiper *et al* (3) demonstrated that the tumor size and depth, and the presence of regional lymph node metastases and positive resection margins are significant risk factors affecting the survival rates of patients with MFH. Due to a low incidence rate of radiation-induced MFH, there has been no large study performing a survival analysis for this malignancy. Certain studies have indicated that following treatment, patients should be followed up closely, with palpation of the regional lymph node beds and strict detection within the radiation field by CT or MRI approximately every 3–6 months for two years, followed by at least annual visits subsequently (23,24). Clearly, the patient of the present study will have to undergo extensive follow-up examinations.

In summary, the present study reports a case of MFH of the upper chest wall that appeared four years after initial radiotherapy for squamous cell carcinoma of the nasopharynx. Additionally, the successful two-step surgery procedure was revealed, which highlighted the prior choice of surgical treatment for this malignant disease. Even so, the importance of the prevention of radiation-induced MFH is emphasized, including the use of proper radiotherapy doses or regions, more advanced radiation techniques and regular follow-up examinations subsequent to irradiation. Additionally, studies on the diagnostic and prognostic characteristics of the malignancy should be pursued. Further studies are required with regard to the assessment of adjuvant therapies as the our knowledge of this disease and its treatment requires enhancement.
